# A multicentric, hyaline vascular variant of Castleman's disease associated with B cell lymphoma: a case report

**DOI:** 10.4076/1757-1626-2-8183

**Published:** 2009-06-10

**Authors:** Mehmet Ali Erkurt, Ismet Aydogdu, Irfan Kuku, Emin Kaya, Bulent Mizrak, Yalcın Basaran

**Affiliations:** 1 Department of Hematology, Inonu University Faculty of MedicineMalatya-Elazig Street, Malatya, 44069Turkey; 2 Department of Pathology, Inonu University Faculty of MedicineMalatya-Elazig Street Malatya, 44069Turkey; 3 Department of Internal Medicine, Gulhane Military Medical AcademyTevfik Saglam Street, Ankara, 06010Turkey

## Abstract

**Introduction:**

Three histological variants (hyaline vascular, plasma cell, and mixed) and two clinical types (localized and multicentric) of Castleman's disease have been described. The risk of progression to lymphoma is higher in multicentric Castleman's disease and is associated with poorer outcomes and higher mortality rate. Multicentric Castleman's disease often requires systemic therapy. Complete resection of the involved node in localized Castleman's disease is curative, with no reported recurrences.

**Case presentation:**

We report a case of a 66-year-old female with systemic symptoms and bilateral cervical lymph nodes which were initially diagnosed as the hyaline vascular variant of Castleman's disease and two years later after the initial diagnosis she was confirmed to B cell lymphoma. Following the treatment with radiation therapy to the cervical area and combination chemotherapy complete response was achieved.

**Conclusion:**

Although it has rarely been reported, the malignant potential of the Castleman's disease must be kept in mind.

## Introduction

Castleman's disease (CD) was firstly defined in 1956 as a mediastinal lymph node hyperplasia consisting of “regressed” germinal center and capillary “proliferation”. It may also be named as mediastinal lymph node tumor, giant lymph node hyperplasia, or angiofollicular lymph node hyperplasia. The etiopathology is unknown [1]. It is classified into three major histopathological subtypes: hyaline vascular variant, plasma cell variant and mixed type. It may also be categorized clinically as localized (unicentric) and multicentric types [2]. The hyaline vascular variant of CD is characterized by a marked increase of abnormal follicles with athrophic germinal centers which are poor in terms of follicular cells and rich of dendritic reticulum cells. Prominent interfollicular vascular proliferation and hyaline material storage are observed in this type. On the other hand, the plasma cell variant of CD has hyperplastic germinal centers. Interfollicular zone is characterized by mature plasma cells, large follicles and rare vascular stroma. 2/3 of the cases are associated with mediastinal and pulmonary involvement, while the other 1/3 with extranodal and non-mediastinal involvement [2,3]. The hyaline vascular variant is seen by 90 percent and the ongoing is mostly asymptomatic. Approximately 10 percent of all cases are of the plasma cell variant, while a small percent had a mixed histological appearance. The plasma cell variant of CD has a more aggressive behavior compared to the hyaline vascular variant. 90 percent of the multicentric cases consist of the plasma cell variant [2]. Multicentric CD is a systemic disease with generalized peripheral lymphadenopathy, hepatosplenomegaly, frequent fevers, night sweats, weight loss, and weakness or fatigue. It is aggressive in behavior and is usually fatal due to fulminant infections or related malignancies, particularly Kaposi's sarcoma and lymphomas [4-6]. Localized CD is most often an isolated benign lymphoproliferative disorder and the vast majority of cases are of the hyaline vascular variant. It is usually asymptomatic and is rarely associated with an increased risk of lymphoma [7]. In this report, we present a rare case of multicentric, hyaline vascular variant of Castleman's disease that transformed to associated with B cell lymphoma.

## Case presentation

**Figure 1.**
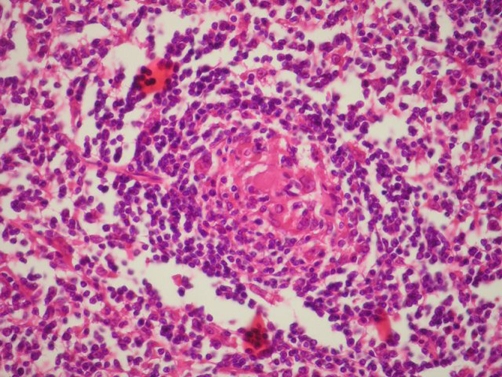
Hyalinized germinal center surrounded with mature lymphoid cells. (Hematoxylin & eosin stain; original magnification x 40).

**Figure 2.**
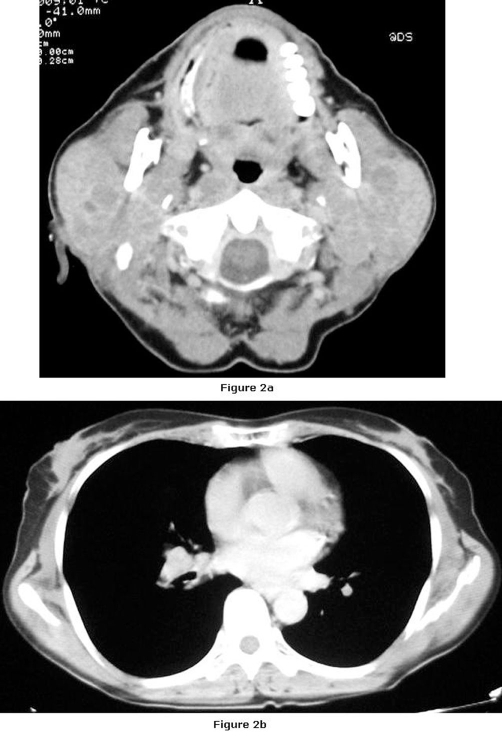
**(A)** Cervical CT of the patient before the treatment. **(B)** Thorax CT of the patient before the treatment.

**Figure 3.**
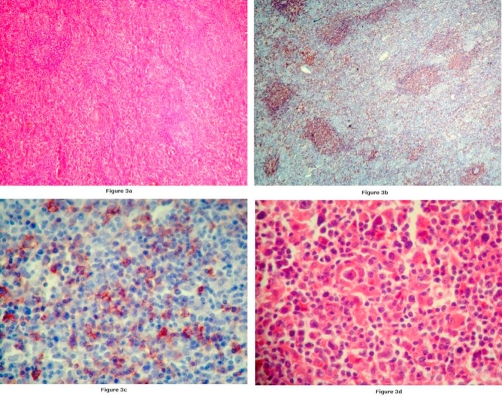
**(A)** Athropic germinal centers and thick walled vessels in lymphoid follicles Parafollicular areas are enlarged. (Hematoxylin & eosin stain; original magnification x 4). **(B)** Immunohistochemically CD20 positive areas 4x. **(C)** Immunohistochemically CD20 positive lymphoid malignant cells 40x. **(D)** A few of malign lymphocytic cells (Hematoxylin & eosin stain; original magnification x 40).

We present a case of a 66-year-old Turkish female with the multicentric, hyaline vascular variant of CD that associated with diffuse large B cell lymphoma. She applied to our center in January 2006, with bilateral cervical masses which have persisted two years since the initial diagnosis and progressively growed. She had dyspnea and pain due to the enlarged lymph nodes. She had also a history of constitutional symptoms such as weight loss and night sweats. Biopsies had been performed form multiple cervical nodes. Pathologic examination of the resected nodes had been reported as reactive lymph node hyperplasia (Figure 1) and she had been followed up without any treatment. Her complaints had persisted and an excisional biopsy of a supraclavicular lymph node had been obtained in March 2005. Pathologic examination of the node had been reported as the hyaline vascular variant of CD. A complete physical examination was performed at presentation which revealed multiple submandibular, pre-postauricular, supraclavicular lymphadenopathies. They were freely movable in the subcutaneous space, non-tender, rubbery and the biggest one was 3 × 2 cm in diameter. On examination, there was no ulceration, retraction, skin dimpling and discharge was not expressed. Otorhinolaryngologic examination was normal and no hepatosplenomegaly was detected. There was bilateral inguinal lymphadenopathy. Cranial nerve examination was normal. Complete blood count, routine biochemical tests and Ig G, A, M, kappa, lambda levels were within normal limits. EBV, HHV8 and HIV serology were negative. In the bone marrow evaluation (aspiration and biopsy) no signs of lymphoma involvement were demonstrated. Bone marrow assessment by flow cytometry revealed neoplastic cells positive for CD19, CD20, CD22, CD45 and CD10 and negative for CD2, CD3, CD5 and CD138. Computed tomographic scan showed the presence of multiple enlarged lymph nodes of the head and neck (parotid, submandibular, anterior and posterior cervical nodes) and cervical lymph node conglomeration in diameter of 3 × 2 cm, which was narrowing the airway at the oropharyngeal level Thoracic CT findings consisted of enlarged, conglomerated mediastinal, prevascular, paratracheal, bilateral hiler and subcarinal lymph nodes and a subpleural node, 1 cm in diameter, at the medio-basal segment of the left lower lobe (Figure 2A,B). Abdominal computed tomography revealed conglomerated lymphadenopaties in both inguinal areas. The involved nodes were resected and they were taken under histopathologic evaluation. Six capsulated lymph nodes were macroscopically observed, the biggest 3 × 2 cm, and the smallest 1 × 1 cm in diameter. Microscopic appearance of paraffin sections showed hyperplastic lymph nodes with atrophic germinal centers. These lymph nodes had a prominent proliferation of hyalinized follicles with marked interfollicular vascular proliferation. Some of the resected nodes were totally ruined. Analysis of cell surface markers on reactive lymph nodes revealed the lack of CD30, CD99, CD4, CD8, CD3, CD5, and also the expression of CD20-CD79a on atypic cells (Figure 3A-D). Both morphologic and immunophenotypic features suggested us associated with CD to B cell lymphoma. The patient was classified as having stage 3B disease. When the diagnosis was established she received radiotherapy (a total dose of 4000 cGy) to cervical area as initial treatment. RT was followed by six cycles of CHOP chemotherapy (Cyclophosphamide 750 mg/m^2^/day, Doxorubicin 50 mg/m^2^/day, Vincristine 1.4 mg/m^2^/day and Prednisone 100 mg/day). Following the treatment with radiotherapy and aggressive combination chemotherapy a durable complete response was achieved. The patient is now in complete remission with a follow-up of 2 years.

## Discussion

CD, firstly described by Benjamin Castleman in 1956, is a lymphoproliferative disorder of unknown etiology [8]. It is also known as angiomatous lymphoid hamartoma, giant lymph node hyperplasia, angiofollicular lymph node hyperplasia or lymphoreticuloma [2,8,9]. The generation of IL-6 in germinal centers of hyperplastic lymph nodes, which results in induction of B-cell differentiation, seems to be implicated in the etiopathogenesis of the disease [4]. Thoracic, cervical, abdominal, and axillary involvements are reported to be 60, 14, 11, 4 percent, respectively [10]. The outcomes of the hyaline vascular variant of CD generally remain favorable. Symptoms associated with the presence of a space-occupying lesion, including cough, dyspnea, dysphagia, hemopthysis, angina, upper respiratory system infections and back pain may be observed [3]. On the other hand, patients with the multicentric, plasma cell variant of CD present with fever, which is nearly universal, as well as night sweats, weight loss, weakness or fatigue and sometimes with polyneuropathies. Additionally, anemia, leucocytosis, hypergammaglobulinemia, hypoalbuminemia, elevated sedimentation rate and increased levels of CRP may also be observed [11]. This case had a two year history of weight loss and night sweating but routine investigations such as complete blood count and biochemical profile were found to be within normal range. It has been shown that there is a strong association between the multicentric, plasma cell variant of CD and HHV-8. It is assumed that this virus increases the risk of extranodal B cell lymphoma and Kaposi's sarcoma by inducing the expression of IL-6 which is found to stimulate vascular endothelial growth factor expression [2,12]. This case, the serologic tests for HIV and HHV-8 infections were negative. Imaging techniques may provide information in identifying and determining the extent of the lymphadenopathy, but have little impact on definite diagnosis. The most critical diagnostic test is an accurate histopathologic evaluation. Immunohistochemical assessment is important in diagnosis. Although the diagnosis can be suggested by routine radiologic studies and fine needle aspiration, this case was confirmed with immunohistochemical assessment of the resected nodes. The available literature suggests that progression of the multicentric, variant of CD to B cell lymphoma is more frequent. Additionally, the progression of hyaline vascular type of CD to malignant lymphoma is very rare and only small number of cases has been identified in the literature [7]. Because of progressive enlargement of the lymph nodes and the persistence of the systemic symptoms, since the initial evaluation of the patient, excisional biopsy was repeated. Histopathologic evaluation of the resected nodes revealed associated with B cell lymphoma. Prognosis of CD strongly depends on the precise histologic subtype, the extent and sites of disease. Mortality rates have been reported to be more than 50 percent in multicentic CD due to infectious complications and related malignancies [9]. The median survival time is 29 months and 26 percent of the patients die in a year after the initial diagnosis. Surgical resection is sufficient in the treatment of localized CD, it generally does not have a role in the treatment of multicentric CD, and therefore a multidiscipliner approach must be provided. Chemotherapy, radiotherapy, interferon alpha and steroids may be used as treatment [2,6,13,14]. At admission to our clinic, the patient presented with night sweats, weight loss and progressively enlarged cervical lymph nodes within a 2-year period. No viral infection was detected. It is difficult to establish an accurate estimate, whether the development of lymphoma occurred de novo or was secondary to CD in this case. Although CD was histopathologic confirmed by lymph node biopsy, we did not perform further immunohistochemical assessment and we do not really know if it was initially associated with lymphoma. Although receiving no treatment over a 2-year period until the day before diagnosis and having less aggressive clinical course, as usually not expected in patients with lymphoma, it is highly likely that Multicentric CD associated with lymphoma. The incidence of lymphoma is increased in patients with CD compared with the general population. In the literature, progression to malignant lymphoma in Multicentric CD associated with HIV is frequent, and within a prospective cohort study of 60 HIV-infected patients with Multicentric CD, and a follow-up period of 20 months, 14 patients (23%) are reported to develop HHV8-associated non-Hodgkin lymphoma. The five-year survival rate in Multicentric CD is 82% and this prognosis appears to be far better than that encountered with malignant lymphomas. Multicentric CD runs a more aggressive course and can progress to non-Hodgkin's lymphoma. Multicentric CD, whether or not HHV8- associated, is generally treated with combination chemotherapy (CHOP) [13,14].

In this case, we assumed malignant lymphoma developed two years later after the diagnosis of the multicentric, hyaline vascular variant of CD. Following the treatment with radiotherapy and combination chemotherapy a durable complete response was achieved.

## Conclusion

The multicentric, hyaline vascular variant of CD has rarely been reported in the literature. Multicentric CD runs a more aggressive course and can progress to non-Hodgkin's lymhoma. Because of its malignant potential, it must be kept in mind as a differential diagnosis in the patients with lymphadenopathic presentations. Follow-up is necessary to detect the malignant lesions. Prompt recognition and therapy is critical for these situations, which may be life-threatening.

## Abbreviations

CD: Castleman's disease
CD: Cluster of Differentiation
CHOP: Cyclophosphamide- doxorubicin-vincristine-prednisone
CRP: C-reactive protein
CT: Computer tomography
EBV: Ebstein Barr Virus
HHV: human herpesvirus
HIV: human immunodeficiency virus
Ig: immunglobuline
